# A systematic review and meta-analysis of gut microbiota in diabetic kidney disease: Comparisons with diabetes mellitus, non-diabetic kidney disease, and healthy individuals

**DOI:** 10.3389/fendo.2022.1018093

**Published:** 2022-10-20

**Authors:** Shisheng Han, Min Chen, Pei Cheng, Zeng Zhang, Yan Lu, Yanqiu Xu, Yi Wang

**Affiliations:** ^1^ Department of Nephrology, Yueyang Hospital of Integrated Traditional Chinese and Western Medicine, Shanghai University of Traditional Chinese Medicine, Shanghai, China; ^2^ Department of Hemodialysis, Lin’an Third People’s Hospital, Hangzhou, Zhejiang, China; ^3^ Department of Endocrine, Yueyang Hospital of Integrated Traditional Chinese and Western Medicine, Shanghai University of Traditional Chinese Medicine, Shanghai, China

**Keywords:** gut microbiota, diabetic kidney disease, diabetes mellitus, systematic review, meta-analysis

## Abstract

**Background:**

Gut microbiota has been reported to play an important role in diabetic kidney disease (DKD), however, the alterations of gut bacteria have not been determined.

**Methods:**

Studies comparing the differences of gut microbiome between patients with DKD and non-DKD individuals using high-throughput sequencing technology, were systematically searched and reviewed. Outcomes were set as gut bacterial diversity, microbial composition, and correlation with clinical parameters of DKD. Qualitative data were summarized and compared through a funnel R script, and quantitative data were estimated by meta-analysis.

**Results:**

A total of 15 studies and 1640 participants were included, the comparisons were conducted between DKD, diabetes mellitus (DM), non-diabetic kidney disease (NDKD), and healthy controls. There were no significant differences of α-diversity between DKD and DM, and between DKD and NDKD, however, significant lower microbial richness was found in DKD compared to healthy controls. Different bacterial compositions were found between DKD and non-DKD subjects. The phylum *Actinobacteria* were found to be enriched in DKD compared to healthy controls. At the genus level, we found the enrichment of *Hungatella*, *Bilophila*, and *Escherichia* in DKD compared to DM, patients with DKD showed lower abundances of *Faecalibacterium* compared to those with NDKD. The genera *Butyricicoccus*, *Faecalibacterium*, and *Lachnospira* were depleted in DKD compared to healthy controls, whereas *Hungatella*, *Escherichia*, and *lactobacillus* were significantly enriched. The genus *Ruminococcus torques group* was demonstrated to be inversely correlated with estimated glomerular filtration rate of DKD.

**Conclusions:**

Gut bacterial alterations was demonstrated in DKD, characterized by the enrichment of the genera *Hungatella* and *Escherichia*, and the depletion of butyrate-producing bacteria, which might be associated with the occurrence and development of DKD. Further studies are still needed to validate these findings, due to substantial heterogeneity.

**Systematic review registration:**

https://www.crd.york.ac.uk/prospero/, identifier CRD42022340870.

## Introduction

Diabetic kidney disease (DKD) or diabetic nephropathy (DN), is one of the most common microvascular complication of diabetes mellitus (DM), characterized by progressive renal impairment and albuminuria (1). The condition is a major cause of chronic kidney disease (CKD) and end-stage kidney disease (ESKD), and is associated with higher risk of cardiovascular events and all-cause mortality in diabetic patients (2). Data from the United States Renal Data System indicated that DKD was the leading attributable cause of ESKD, accounting for 46.6% in 2019 (3). Numerous efficacious therapies have been successfully administrated for DKD and have shown renal benefits, such as renin-angiotensin system (RAS) inhibitors, sodium-glucose cotransporter-2 inhibitors, incretin-based therapeutic agents, and finerenone (4); however, substantial residual risk of irreparable renal failure remains (5). Given that the pathological mechanism of DKD has not yet been elucidated, more understanding of the pathogenesis of DKD is urgent for its prevention and treatment. Gut microbiome is relatively stable and participates in various physiological processes (6). However, gut dysbiosis, characterized by imbalance of gut bacterial composition, was found to be associated with the onset and progression of numerous chronic diseases (7). Recently, mounting evidence supports the important role of gut microbiota and their metabolites in diabetes and DKD (8). Excess acetate produced by gut dysbiosis has been shown to be involved in renal injury by activating intrarenal RAS (9), and contributed to tubulointerstitial injury through regulating cholesterol homeostasis *in vivo* and *in vitro* (10). Gut microbiota depletion mediated by antibiotic and faecal microbiota transplantation attenuated glomerular injury and stabilized metabolic homeostasis (11). Dietary fiber showed renoprotective effects of relieving albuminuria and attenuating glomerular injury and interstitial fibrosis, through reshaping gut microbial ecology and promoting the expansion of short-chain fatty acid (SCFA)-producing bacteria in diabetic mice (12). Patients with DN receiving supplementation of probiotics for 12 weeks showed significantly lower serum creatinine and albuminuria than those receiving placebo (13, 14). Given the potential pathogenic role of intestinal dysbiosis in DKD according to recent evidence, characterizing the gut microbiota in DKD might be beneficial for formulating therapeutic strategy. Previous investigations have reported the existence of gut dysbiosis in patients with DKD compared to healthy volunteers, including the changes bacterial diversity and alterations of microbial composition, however, their findings were inconsistent (15). Additionally, the differences of gut microbiota between DKD and DM or non-diabetic kidney disease (NDKD) were also not determined. This systematic review was designed to compare the differences of microbial diversity and bacterial composition between patients with DKD and non-DKD individuals, aiming to characterize the alterations of gut bacteria in DKD and provide potential microbiota targets for the intervention of DKD.

## Materials and methods

### Registration and statement

This systematic review was pre-registered in International Prospective Register of Systematic Reviews (PROSPERO, CRD42022340870) and performed in accordance with the guidelines of Preferred Reporting Items for Systematic Reviews and Meta-Analyses (PRISMA) (Checklist, **Supplementary Table 1**) (16).

### Search strategy

The literature search was conducted on PubMed, Embase, Web of Science, China national knowledge infrastructure, Cochrane Library, and *ClinicalTrials.gov*, from inception until 3 July 2022. Eligible studies comparing gut microbiota between patients with DKD and non-DKD persons were retrieved using the search terms with DKD, gut microbiota, and their relevant keywords. Our detailed searching strategies for each database is detailed in **Supplementary Table 2**.

### Eligible criteria and outcomes

Studies comparing the diversity and/or composition of gut microbiota between patients with DKD and non-DKD individuals using high-throughput sequencing technology, were included. The inclusion criteria and exclusion criteria according to the PICOS principle are shown in **Table 1**. The primary outcome was gut microbial diversity, and the secondary outcomes were gut microbial composition and the correlations between clinical parameters of DKD and specific bacteria. In this review, we compared α-diversity and β-diversity between different groups. Bacterial a-diversity was evaluated by observed species/Chao1/ACE-based richness index and Shannon/Simpson-based community diversity index. β-diversity represents the differences of gut microbial structure between DKD and non-DKD individuals.

**Table 1:** Eligible criteria based on PICOS.

	Inclusion criteria	Exclusion criteria
Participant	Patients with DKD diagnosed clinically or biopsy-proven DN	Patients receiving dialysis; patients with diabetes and other chronic kidney disease
Exposure	DKD/DN	–
Comparator	DKD vs. Non-DKD, including diabetes mellitus, non-diabetic kidney disease, and healthy controls	–
Outcomes	Gut microbial diversity and composition	Insufficient data for analysis; not high-throughput sequencing technology for detecting gut microbiome
Study design	Observational study	–

DKD, diabetic kidney disease; DN, diabetic nephropathy.

### Study selection, data extraction, and quality assessment

After removal of duplicates, two reviewers screened titles and abstracts of the retrieved records independently (S.H. and P.C.), and disagreements were solved by discussing with a third researcher (Y.X.). The full texts were screened for eligible studies by Y.L. and Y.W. For each included study, two reviewers (Z.Z. and M.C.) extracted the following data independently: author, publication year, country, study design, diagnostic criteria of DKD, characteristics of all groups, including sample size, age, sex, matched factors, serum creatinine, estimated glomerular filtration rate (eGFR), urinary albumin-creatinine ratio (UACR), and urinary total protein (UTP), stool sample collection and storage, DNA extraction method, sequencing platform, bioinformatics pipelines, and outcomes. Methodological quality was assessed using the Newcastle-Ottawa Scale (NOS) for case-control study and the modified version for cross-sectional study. The NOS scale has three domains for evaluation: selection, comparability, and exposure/outcome, maximizing 9 scores for case-control study and 7 scores for cross-sectional study. A total score of ≥ 7 for case-control studies and ≥ 4 scores for cross-sectional studies were considered as high quality (17).

### Statistical analysis

The quantitative and qualitative data of gut microbiota diversity and relative abundance between DKD and non-DKD individuals reported in each study were record, and were synthesized by qualitative summary and meta-analysis, respectively. For qualitative analysis, the results of each prespecified outcome were summarized and presented as stacked histograms. A funnel R script was adopted to explore differential bacteria between different groups at the significance levels of 80% and 95%, through calculating a binomial Poisson distribution score2 (18). For meta-analysis, standardized mean difference (SMD) and 95% confidence intervals (CIs) were calculated to evaluate the differences in diversity indices and relative abundances of gut bacteria between DKD and non-DKD groups. Heterogeneity was quantified using Cochrane *I* ^2^ test, which was considered significant when *I* ^2^ > 50% (19). Meta-analysis was then conducted to estimate pooled SMD using a fixed-effects model or a random-effects model according to heterogeneity. Sensitivity analysis and subgroup analysis were performed according to different inclusion criteria of DKD. All the statistical processes and results visualization were conducted by Stata (version 14.0), RStudio (Open source, version 2021.9.2 + 382), and GraphPad Prism (version 8.0).

## Results

### Study characteristics

According to our retrieval strategy, a total of 8618 records were searched from the electronic databases and registers. After removing duplicates and screening titles and abstracts, 8569 publications were excluded. Ultimately, 15 studies were selected according to the inclusion criteria and exclusion criteria during full-text screen. The study selection process and reasons for exclusion are shown in **Supplementary Figure 1**.

The characteristics of included studies in this review are presented in **Table 2**, including 15 cross-sectional studies published from 2019 to 2022 (20–34). One study was conducted in Denmark (22), and the other fourteen studies were completed in China. Two studies included patients with biopsy-proven DN (20, 31), and the remaining 13 studies enrolled patients with DKD who were diagnosed clinically. Eight studies compared the differences of gut microbiota among patients with DKD, patients with DM, and healthy volunteers (20, 21, 24, 25, 28, 30, 32, 34), four studies reported the differences of intestinal microbiota between DKD and healthy controls (22, 26, 29, 31), one study conducted the comparison between DKD and type 2 DM (33), and the other two studies analyzed the differences of gut bacteria between patients with DKD and those with NDKD (23, 27). All the included studies stated that they have excluded subjects with gastro-intestinal or systemic diseases known to affect gut microbiota, and those taking antibiotics or prebiotics/probiotics within 1 to 3 months before enrollment. According to the included studies, 830 fecal specimens were collected from patients with DKD, 514 from healthy volunteers, 256 from diabetic individuals, and 40 from patients with NDKD. All the enrolled studies reported that fresh stool samples were collected and stored at −80°C until DNA extraction, and 16S ribosomal gene amplicon sequencing was adopted for gut microbiota analysis. The amplified region was V3-V4 in eight studies (20, 23, 25–27, 29, 32, 34), V3 in one study (24), and V4 in four studies (21, 22, 28, 30), two studies did not report the amplified region (31, 33). Illumina sequencing platform was adopted in 14 studies, while only one study used the Ion S5TM platform (32).

**Table 2:** Characteristics of the included study.

Study	Location	Comparisons	Eligible criteria of DKD/DN	Case [n (female%, age)]	Control [n(female%, age)]	Matched factors	Sequencing platform (Region)	Database	Outcomes	Modified NOS score
Tao 2019 (20)	Guangdong, China	DN vs. T2DM vs. HC	Biopsy-proven DN, eGFR ≥ 60 mL/min/1.73 m^2^ and UACR ≥ 30 mg/g	14 (36%), 52.93 ± 9.98	T2DM: 14 (36%), 53.29 ± 9.00; HC: 14 (36%), 52.86 ± 9.91	Age, sex, BMI	Illumina MiSeq (V3-V4)	RDP, Silva	α-diversity; β-diversity; microbial composition; clinical correlation	7
Bao 2019 (21)	Sichuan, China	DKD vs. T2DM vs. HC	DM complicated with massive proteinuria; or with DR and CKD; microalbuminuria in T1DM of more than 10 years	25 (36%), 63.7 ± 13.3	T2DM: 30 (54%), 62 ± 13.3; HC: 30 (47%), 60.2 ± 9.7	Age, sex	Illumina TruSeqTM (V4)	Greengenes	α-diversity; β-diversity; microbial composition	7
Winther 2020 (22)	Copenhagen, Denmark	T1DKD vs. HC	T1DM with eGFR ≥ 15 mL/min/1.73 m^2^ and excluded non-diabetic kidney disease	161 (42%), 60 ± 10	50 (44%), 59 ± 13	Age, sex, BMI	Illumina HiSeq2500 (V4)	Dada2 R package	α-diversity; β-diversity; microbial composition; clinical correlation	7
Yu 2020 (23)	Henan, China	DKD vs. MN	Diabetes more than 5 years with UACR ≥ 30 mg/g and presence of DR	129 (36%), 56 (49, 65)	142 (36%), 49 (43, 56)	Sex	Illumina MiSeq (V3-V4)	RDP, Silva	α-diversity; β-diversity; microbial composition	6
Feng 2020 (24)	Sichuan, China	DKD vs. T2DM vs. HC	Clinically diagnosed DKD and not on dialysis	57 (41%), 55.23 ± 11.21	T2DM: 68 (47%), 54.36 ± 11.12; HC: 36 (42%), 54.84 ± 11.17	Age, sex	Illumina HiSeq 2500 (V3)	NR	β-diversity; microbial composition	6
Chen 2021 (25)	Beijing, China	DKD vs. DM vs. HC	DKD with UAER ≥ 30 mg/24h or UACR ≥ 30 mg/g	60 (32%), 60.53 ± 9.62	DM: 20 (40%), 55.2 ± 14.77; HC: 20 (60%), 55.15 ± 13.77	Age, BMI, diet	Illumina MiSeq (V3-V4)	RDP, Silva	α-diversity; microbial composition; clinical correlation	6
Du 2021 (26)	Tianjin, China	DKD vs. HC	DN stage 3 or 4, without detailed criteria, not uremia	43 (26%), 60.86 ± 5.69	37 (33%), 61.78 ± 6.40	Age, sex, BMI	Illumina MiSeq (V3-V4)	RDP, Silva	α-diversity; β-diversity; microbial composition	6
Sun 2021 (27)	Shandong, China	DKD vs. NDKD vs. HC	Clinically diagnosed DKD	25 (36%), 62.52 ± 13.61	NDKD: 40 (23%), 53.98 ± 13.81; HC:24 (34%), 56 ± 9	Age, sex	Illumina MiSeq (V3-V4)	RDP, Silva	α-diversity; β-diversity; microbial composition	6
Song 2021 (28)	Inner Mongolia, China	DKD vs. T2DM vs. HC	Diagnosed DKD	20 (40%), 58.2 ± 9.4	T2DM: 20 (50%), 54.1 ± 13.5 HC: 20 (55%), 50.2 ± 12.6	Age, sex	Ilumina, NovaSeq PE250 (V4)	Dada2 R package	α-diversity; β-diversity; microbial composition; clinical correlation	6
Zhang 2021 (29)	Henan, China	DKD/DN vs. HC	Proteinuria or renal impairment caused by diabetes, and other kidney diseases were excluded, meeting one of the following conditions: UACR ≥ 30mg/g or UAER ≥ 30mg/24h or eGFR ≤ 60mL/min/1.73 m^2^ or biopsy-proven DN	180 (38%), 55.27 (26-87)	179 (42%), 52.05 (39-69)	SCr	Illumina MiSeq (V3-V4)	RDP	α-diversity; β-diversity; microbial composition; clinical correlation	6
Chu 2021 (30)	Zhejiang, China	T2DKD vs. T2DM vs. HC	DKD with UACR ≥ 30 - 300mg/g or UAER 20-200 ug/min	47 (45%), 69.06 ± 11.23	T2DM: 53 (42%), 68.47 ± 10.82 HC: 42 (43%), 67.11 ± 9.26	Age, sex	Ilumina HiSeq (V4)	NCBI-BLAST	α-diversity; microbial composition	6
Xin 2021 (31)	Shanxi, China	DN vs. HC	Biopsy-proven DN	20 (50%), 55.1 ± 13.83	20 (50%), 50.9 ± 9.49	Age, sex	Ilumina Novaseq6000 (NR)	HUMAnN3	α-diversity; β-diversity; microbial composition; clinical correlation	7
Cai 2022 (32)	Zhejiang, China	DKD vs. T2DM vs. HC	DKD diagnosed clinically: UAER >300 mg/24h and presence of DR, and excluded other kidney diseases	31 (26%), 61.35 ± 10.04	T2DM: 32 (32%), 56.34 ± 10.79; HC: 34 (65%), 56.12 ± 8.11	Age	Ion S5TM (V3-V4)	Ion 530TM Chip	α-diversity; β-diversity; microbial composition	6
He 2022 (33)	Shanxi, China	DKD vs. T2DM	Diagnosed DKD stage 3 or4, presenting normal renal function and UACR > 30 mg/g, renal impairment due to other causes was excluded.	10 (10%), 56.00 ± 14.97	10 (20%), 64.90 ± 7.37	Age, sex, BMI	Illumina HiSeq4000 (NR)	Non-Redundant	β-diversity; microbial composition; clinical correlation	7
Yang 2022 (34)	Guizhou, China	DKD vs. T2DM vs. HC	T2DM with ACR > 265 mg/g or UAER > 300 mg/24h; and/or DR with ACR 22 (male, 31 female) - 265 mg/g; or UAER 30 - 300 mg/24h and/or eGFR < 60 min/mL.	8 (50%), 58.75 ± 7.40	T2DM: 9 (56%), 57.67 ± 4.61; HC: 8 (50%), 57.13 ± 2.8	Age, sex, BMI	Illumina MiSeq (V3-V4)	Greengenes	α-diversity; β-diversity; microbial composition	7

DKD, diabetic kidney disease; DN, diabetic nephropathy; T2DM, type 2 diabetes mellitus; HC, healthy controls; eGFR, estimated glomerular filtration rate; UACR, urinary albumin-creatinine ratio; UAER, urinary albumin excretion rate; NOS, Newcastle-Ottawa Scale; DR, diabetes retinopathy; CKD, chronic kidney disease; BMI, body mass index; SCr, serum creatinine; NR, not reported; RDP, ribosomal database project.

Six studies were awarded seven scores according to the modified NOS scale for cross-sectional studies, because of adequate selection for subjects, sufficient ascertainment of outcome, and controls of at least two confounding factors (20–22, 31, 33, 34). Three studies were assessed for six scores, because there were only one factor were matched between cases and controls (23, 29, 32). Six studies were given six scores, due to the absence of detailed diagnostic criteria of DKD (24–28, 30).

### Bacterial diversity

The purpose of this review was to explore the alterations of gut microbiota in patients with DKD. According to the existing evidence, the comparisons of intestinal bacteria were carried out between DKD and DM, DKD and healthy control, and DKD and NDKD, respectively.

**Figure 1:**
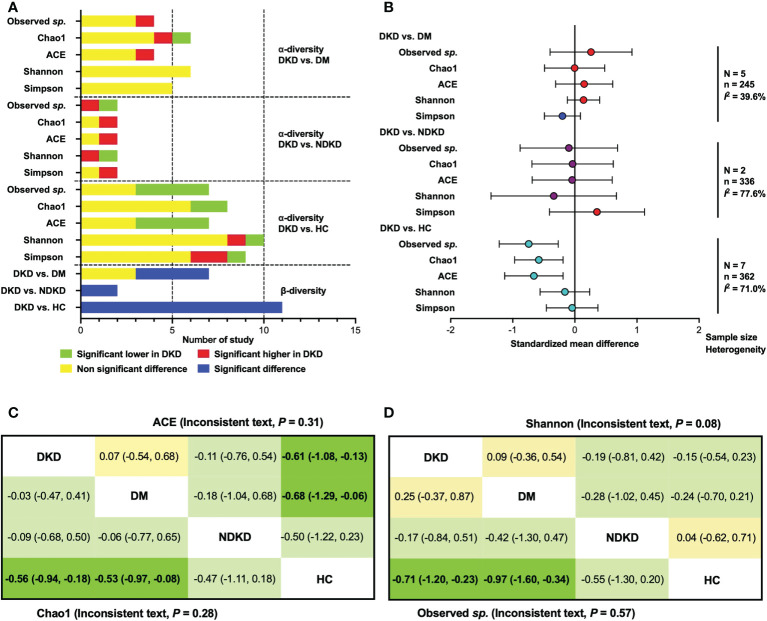
Qualitative analysis and meta-analysis for α- and β-diversity. **(A)** Qualitative comparisons for α- and β- diversity; **(B)** Meta-analysis for α-diversity indices. **(C)** Network meta-analysis for ACE and Chao1 index; **(D)** Network meta-analysis for Observed sp. and Shannon index. Data are shown as standardized mean difference (95% confidence interval). The estimate is for the column-defining treatment compared to the row-defining treatment. Statistical significance is defined as 95% CIs that do not overlap zero (bold text). DKD, diabetic kidney disease; DM, diabetes mellitus; HC, healthy controls; NDKD, non-diabetic kidney disease; N, number of study.

The qualitative comparisons of microbial diversity indices between patients with DKD, diabetes individuals, and healthy controls are presented in **Figure 1A**. Three of four studies reported non-significantly changes of observed species (21, 25, 28) and ACE index (20, 30, 34) between the DKD and DM groups, while Tao et al. (20) and Cai et al. (32) reported an increased indices of observed species and ACE in patients with DKD, respectively. The Chao1 index was found to be significantly higher in patients with DKD than diabetes patients in one study (32), lower in one study (28), and not significantly changed in four studies (20, 25, 30, 34). Six (20, 25, 28, 30, 32, 34) and five (20, 28, 30, 32, 34) studies reported that there were no significant differences between DKD and DM in Shannon and Simpson index. Two studies reported the differences of gut microbiome in α-diversity between DKD and NDKD patients (23, 27). Opposite results were shown in the observed species and Shannon index. For Chao1, ACE and Simpson index, one study suggested that they were significantly higher in patients with DKD than in those with NDKD, while another study showed non-significant differences.

Compared with healthy controls, significant lower observed species (21, 26–28) and ACE index (20, 26–28) of gut microbiome in patients with DKD were found in four studies, whereas other three studies reported unchanged proportion (20, 22, 29, 30, 32, 34). Two (26, 28) and six studies (20, 25, 27, 30, 32, 34) reported significantly lower and non-significant alterations of the Chao1 index in DKD patients compared to healthy volunteers, respectively. Shannon index was shown to be significantly higher in DKD patients in one study (31), lower in one study (27), and not changed in eight studies compared to healthy participants (20, 22, 25, 26, 28, 30, 32, 34). For the Simpson index, the number of studies reporting a significant increase (27, 29), a significant decrease (32), and non-significant change in DKD groups compared to healthy groups (20, 22, 26, 28, 30, 34), were 2, 1, and 6, respectively.

Based on the available data of α-diversity index, we conducted a quantitative meta-analysis (**Figure 1B**). The results showed that there were no statistical differences in α-diversity indices of gut bacteria between DKD and DM patients, as well as those between DKD and NDKD individuals. Compared to healthy volunteers, patients with DKD showed significantly lower microbial richness index (Observed sp., SMD = -0.74, 95%CI -1.22, -0.27, *I* ^2^ = 68.5%; ACE, SMD = -0.66, 95%CI -1.13, -0.19, *I* ^2^ = 67.1%; Chao1, SMD = -0.58, 95%CI -0.97, -0.19, *I* ^2^ = 67.1%), whereas no significant differences were found in Shannon and Simpson index. Considering that the comparisons were conducted among multiple groups, and the tests for subgroup differences were significant in microbial richness indexes (**Supplementary Figure 2**), we further performed a random-effects network meta-analysis for α-diversity utilizing previously reported routines (35). No inconsistency was found in the α-diversity indexes, except Simpson index, which showed significant inconsistency (*P* = 0.02). The results of network comparisons agreed with the above findings, involving observed species, Chao1, ACE, and Shannon index. Additionally, we found that patients with DM also showed lower microbial richness than healthy subjects (**Figures 1C, D**).

Four studies reported significant differences of β-diversity between DKD and DM (20, 32–34), while three studies showed no significant changes (21, 24, 28). Whether compared with patients with NDKD (23, 27) or healthy controls (20–22, 24, 26–29, 31, 32, 34), significant differences in β-diversity were observed in patients with DKD, indicating fecal microbial alterations in DKD.

### Microbial composition at phylum level

**Figure 2:**
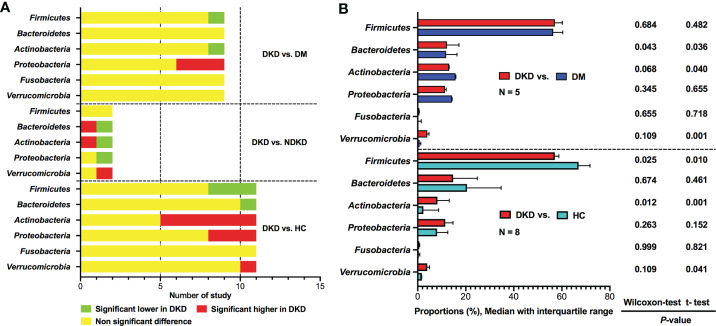
Qualitative and quantitative analysis of gut microbiota at the phylum level. **(A)** Qualitative comparisons at the phylum level; **(B)** Comparisons of average abundances at the study level for bacterial phylum. DKD, diabetic kidney disease; NDKD, non-diabetic kidney disease; DM, diabetes mellitus; HC, healthy controls; N, number of study.

Six phyla were reported dominating the gut microbiota, including *Firmicutes*, *Bacteroidetes*, *Actinobacteria*, *Proteobacteria*, *Fusobacteria*, and *Verrucomicrobia* (**Figure 2A**). Compared to DM group, *Firmicutes* and *Actinobacteria* were found to be depleted in DKD group in one study (20), whereas eight studies reported non-significant differences (21, 24, 25, 28, 30, 32–34). The relative abundances of *Proteobacteria* were shown to be enriched in patients with DKD compared to diabetic persons in three studies (20, 32, 33), while six studies did not find any difference between the two groups (21, 24, 25, 28, 30, 34). The proportions of *Bacteroidetes*, *Fusobacteria*, and *Verrucomicrobia* did not show differences between DKD and DM according to the results of nine studies (20, 21, 24, 25, 28, 30, 32–34). Only two studies compared gut microbiota between DKD and NDKD at phylum level, their results showed that the abundances of *Firmicutes* were similar between the two groups, while the comparisons of *Bacteroidetes*, *Actinobacteria*, *Proteobacteria*, *Fusobacteria*, and *Verrucomicrobia* showed inconsistent results (23, 27).

Eleven studies reported the relative abundances of bacterial phyla between patients with DKD and healthy controls (20, 21, 24–30, 32, 34). Three studies showed decreased abundances of *Firmicutes* in DKD (21, 28, 32), whereas eight studies reported non-significant differences between patients with DKD and healthy volunteers (20, 24–27, 29, 30, 34). For *Bacteroidetes*, only one studies found that it was depleted in DKD group (29), while ten studies showed that the *Bacteroidetes* taxa was not statistically different between DKD patients and healthy individuals (20, 21, 24–28, 30, 32, 34). *Actinobacteria* was found to be higher in patients with DKD than those in healthy controls in six studies (24–27, 29, 34), however, five studies indicated non-significant differences (20, 21, 28, 30, 32). Regarding *Proteobacteria*, three studies supported increased abundances in DKD (27, 29, 32), while the remaining eight studies did not find differences between DKD and healthy controls (20, 21, 24–26, 28, 30, 34). None of the included studies reported differences in *Fusobacteria* between DKD and healthy controls. *Verrucomicrobia* was reported to be enriched in patients with DKD in one study (29), however, no significant changes were observed in ten studies (20, 21, 24–28, 30, 32, 34).

Due to the limited data, we can only calculate the differences in the average abundances of bacterial phyla between DKD and non-DKD individuals at the study level (**Figure 2B**). Compared with diabetic population, patients with DKD might have mildly increased taxa of *Bacteroidetes*. The average abundance of *Firmicutes* was found to be lower in patients with DKD than that in healthy controls, whereas *Actinobacteria* was significantly enriched in DKD patients, which was consistent with the results of qualitative analysis.

### Microbial composition at genus level

**Figure 3:**
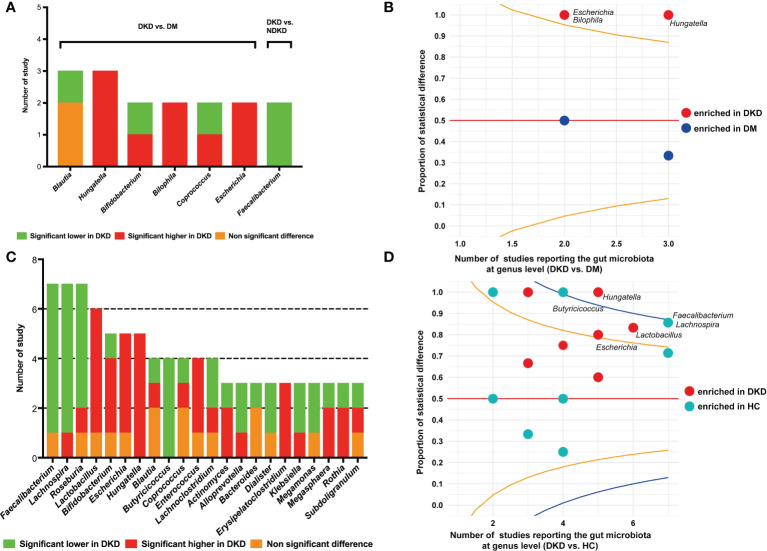
Comparisons of gut microbiota at the genus level. **(A)** The genera reported to be statistically different between DKD and DM, and between DKD and NDKD; **(B)** The funnel plot conducted between DKD and DM, specified score2 confidence limits were showed at 80% (orange line) and 95% (blue line); **(C)** The genera reported to be statistically different between DKD and HC; **(D)** The funnel plot conducted between DKD and HC. DKD, diabetic kidney disease; NDKD, non-diabetic kidney disease; DM, diabetes mellitus; HC, healthy controls.

Eight studies reported the differences of gut bacteria between DKD and DM at the genus level (20, 21, 24, 25, 28, 32–34). The genera that were reported to be statistically different between the two groups in two or more studies are presented in **Figure 3A**. *Hungatella* was shown to be enriched in DKD compared to DM in three studies (20, 28, 32), *Bilophila* and *Escherichia* were found to have higher proportions in DKD patients in two studies (20, 33). The proportion of studies reporting significantly higher or lower abundances of specific genera were compared using a funnel R script, which also suggested that the genera *Hungatella*, *Bilophila*, and *Escherichia* might be the differential bacteria between DKD and DM (**Figure 3B**). When comparing the genera between DKD and NDKD, we found that only *Faecalibacterium* had consistent results in the two studies, that is, it was depleted in DKD patients (**Figure 3A**) (23, 27). Twelve studies presented the comparisons of gut microbiome between DKD and healthy individuals at the genus level (20–22, 24–29, 31, 32, 34). *Faecalibacterium* (21, 22, 26, 27, 29, 31), *Lachnospira* (21, 22, 25, 27, 31, 32), *Roseburia* (21, 25–27, 31), and *Butyricicoccus* (22, 26, 27, 32) were reported to be depleted in DKD in at least four studies, whereas *Hungatella* (20, 22, 28, 31, 32), *Lactobacillus* (21, 22, 26, 27, 29), and *Escherichia* (20, 27, 29, 31) were found to be enriched in DKD in five or four studies (**Figure 3C**). The funnel plot indicated that the genera *Hungatella* were enriched in DKD, whereas *Butyricicoccus*, *Faecalibacterium*, and *Lachnospira* were depleted (**Figure 3D**).

**Figure 4:**
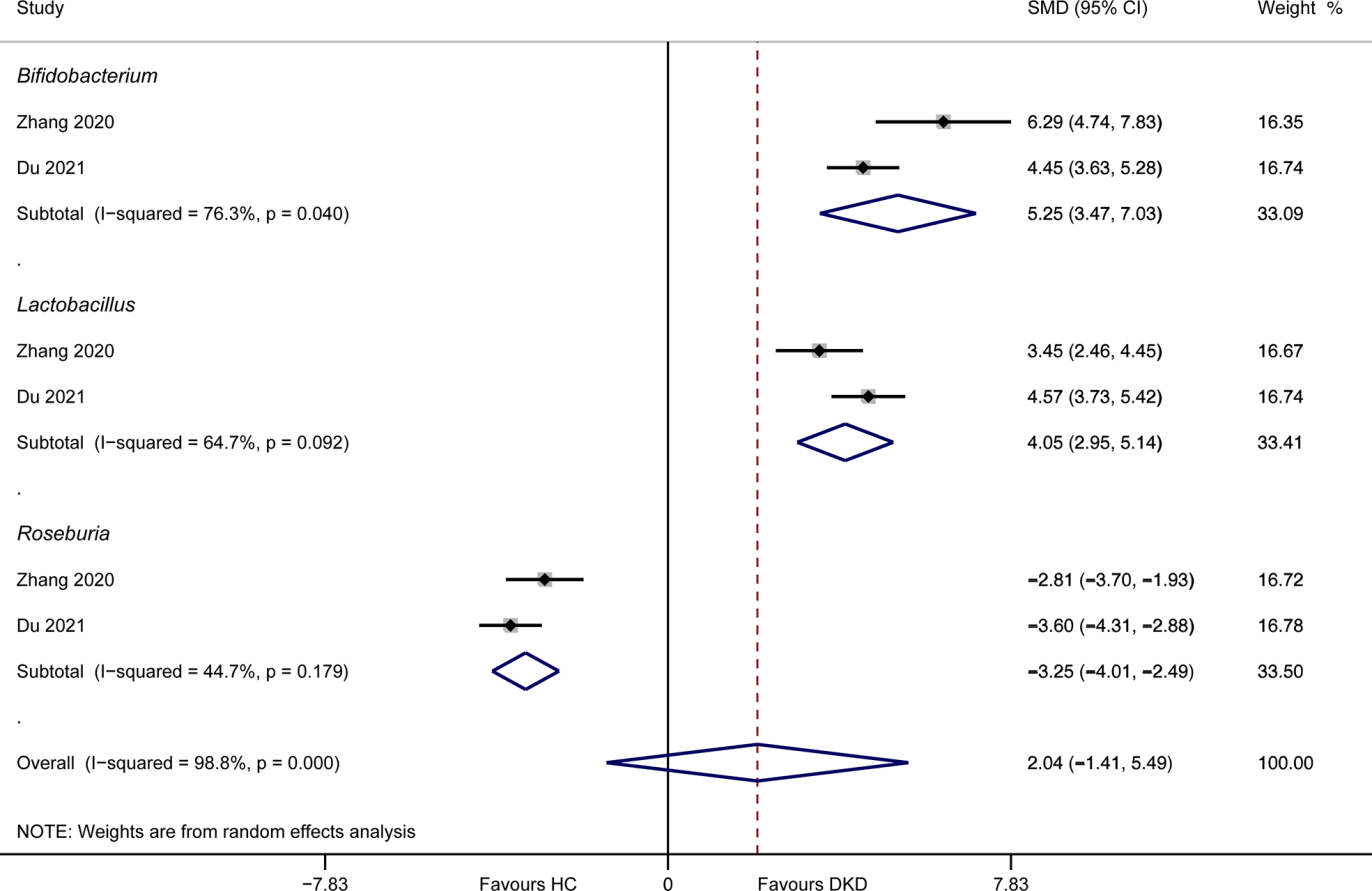
Meta-analysis of the genera between DKD and HC. DKD, diabetic kidney disease; HC, healthy controls; SMD, standardized mean difference; CI, confidence interval.

Only two studies detailed the abundances of gut microbiota between DKD patients and healthy volunteers at genus level. Meta-analysis suggested that *Bifidobacterium* (SMD = 5.25, 95%CI 3.47, 7.03, *I* ^2^ = 76.3%) and *Lactobacillus* (SMD = 4.05, 95%CI 2.95, 5.14, *I* ^2^ = 64.7%) had higher relative proportion in DKD patients compared to healthy persons, while *Roseburia* (SMD = -3.25, 95%CI -4.01, -2.49, *I* ^2^ = 44.7%) was enriched in healthy volunteers (**Figure 4**). The results of *Lactobacillus* and *Roseburia* were consistent with that from qualitative analysis. However, this result should be interpreted with caution, due to the limited data and substantial heterogeneity.

### Correlation of gut microbiota and clinical parameters of DKD

**Figure 5:**
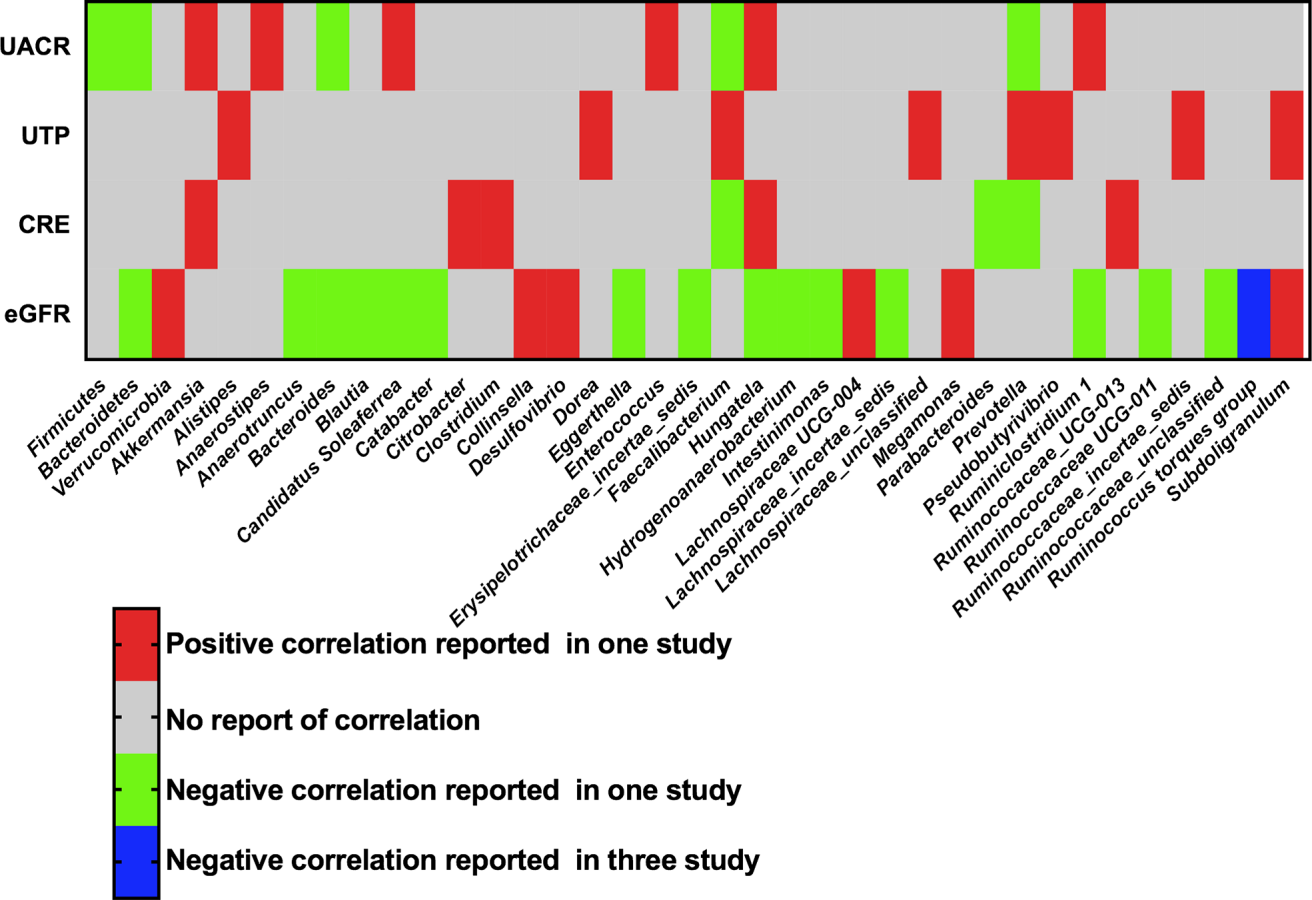
Correlation of gut microbiota and clinical parameters of DKD. UACR, urinary albumin-creatinine ratio; UTP, urinary total protein; CRE, creatinine; eGFR, estimated glomerular filtration rate.

The phyla and genera of gut bacteria with statistical correlation with clinical parameters of DKD were recorded, including UACR, UTP, eGFR, and serum creatinine. Three phyla and thirty-four genera were reported to have a positive or negative association with proteinuria or renal function in at least one study (**Figure 5**). In particular, Three studies supported a negative correlation between the genus *Ruminococcus torques group* (*R. torques*) and eGFR in patients with DKD. Two studies reported that *Hungatella* was positively correlated with serum creatinine and negatively correlated with eGFR, suggesting the harmful effect of *Hungatella* on aggravating kidney injury of DKD (28, 31).

### Sensitivity analysis and subgroup analysis

Considering that the definition of DKD was not consistent across the enrolled studies, involving clinically diagnosed DKD and biopsy-proven DN, we conducted sensitivity analysis and subgroup analysis to test the stability of the results and compare the differences of gut bacteria between different inclusion criteria. When biopsy-proven DN was excluded, the results based on patients with clinically diagnosed DKD were consistent with the findings from the qualitative and quantitative analyses of all the included 15 studies (**Supplementary Figures 3A–C**, **E, F**).

The subgroup of biopsy-proven DN consisted of two studies and 82 participants (20, 31). Detailed α-diversity index was reported in one study (20), indicating higher observed species in biopsy-proven DN than those in DM, and a lower ACE index in DN group compared to healthy controls (**Supplementary Figure 3D**). At the genus level, three genera were found predominantly in biopsy-proven DN versus DM, including *Hungatella*, *Escherichia*, and *Bilophila.* Moreover, *Hungatella* and *Escherichia* were still identified to be enriched in DN group when compared to healthy controls (**Supplementary Figures 3G, H**). These changes in bacterial composition were consistent with the findings from clinically diagnosed DKD, as well as the results from all of the 15 studies, suggesting the potential important roles of *Hungatella* and *Escherichia* in DKD.

## Discussion

Accumulating evidence has demonstrated that alterations of composition and function in gut microbiota were correlated with increased risk of the occurrence and development of diabetes and its associated complications (36). This review was designed to comprehensively characterize the alterations of gut microbiome in DKD, by comparing with diabetes, NDKD, and healthy controls. A total of 15 cross-sectional studies and 1640 participants were included for comparison. There was no significant difference in the α-diversity of gut microbiota between DKD and diabetes subjects, as well as the comparison between DKD and NDKD. Lower microbial richness indices were found in DKD patients compared to healthy volunteers. Unlike α-diversity, β-diversity analysis suggested significant microbial differences between DKD and NDKD and healthy controls; four of seven studies showed significant differences in β-diversity between DKD and DM individuals. At the phylum level, *Actinobacteria* was found to be enriched in DKD compared to healthy controls, however, no significant difference was found when comparing with DM. *Actinobacteria* was closely related to the metabolism of trimethylamine-N- oxide (TMAO), high levels of circulating TMAO were demonstrated to contribute to renal dysfunction through promoting inflammation (37), oxidative stress, and fibrosis (38). Patients with DKD had a significantly higher level of TMAO than those with diabetes, moreover, TMAO also showed positive correlation with UACR (34). At the genus level, *Hungatella*, *Bilophila*, and *Escherichia* showed higher abundances in DKD compared to DM, and *Faecalibacterium* was found to be depleted in DKD compared to NDKD. The genera *Hungatella*, *Bilophila*, and *Escherichia* are all gram-negative, recognized by their pathogenic and infectious potential, since many members are opportunistic pathogens that induce inflammation and disrupt gut barrier function (39–41). Interestingly, patients with type 2 diabetes receiving empagliflozin showed similar gut microbiota alterations with our findings, accompanying with improved glucose metabolism and decreased interleukin-6 (IL-6), that is, depleted taxa of the harmful bacteria of *Escherichia*, *Bilophila*, *and Hungatella*, and enrichment of SCFA-producing bacteria, such as *Roseburia* and *Faecalibacterium* (42). *Hungatella* was reported as a TMAO-producer (41), whereas *Bilophila* is a sulfate-reducing bacteria, which have pro-inflammatory effects and have been shown to be associated with a variety of inflammatory or immune diseases, such as diabetes and metabolic syndrome (43). The genus *Escherichia* was found to be enriched in the stool samples of patients with DKD compared to diabetic persons and healthy volunteers in this review. This findings have also been validated in cohorts of CKD, *Escherichia* was identified as the biomarker for the advanced CKD, and the abundance was positively correlated with CKD stages (44). It is documented that *Escherichia* can metabolize tryptophan into indole, which can be converted into indoxyl sulphate (IS) and Kynurenine, and then participate the process of renal impairment (45). IS and Kynurenine have been proved to have renal injury effects, such as promoting endothelial dysfunction (46), inducing tubulointerstitial injury (47), and aggravating renal oxidative stress and inflammation (48). Serum levels of IS and Kynurenine were shown to be positively associated with the progression of DKD (49, 50). *Escherichia* are also conditional pathogens that can enhance gut infiltration through penetrating the intestinal epithelial barrier and aggravate gut leakiness, resulting in the escape of pathogenic and commensal bacteria and subsequent immune responses (51). The enrichment of *Hungatella*, *Escherichia*, and *Lactobacillus* were found in patients with DKD compared to healthy controls, whereas decreased proportions of the genera *Butyricicoccus*, *Lachnospira*, *Faecalibacterium*, and *Roseburia* were indicated according to the qualitative and quantitative analyses. These four genera are butyrate-producing bacteria (52–54), and have been reported diversified renoprotective effects for DKD *in vivo* and *in vitro*, such as improving intestinal barrier function (55), attenuating fibrosis and collagen deposition, inhibiting inflammation (56), and ameliorating TGF-β1-induced fibrogenesis, apoptosis and DNA damage in the diabetic kidney (57). *Lactobacillus* have been used widely in foods and probiotic products and showed beneficial effects (58), however, upregulated inflammatory cytokines were also significantly increased in *Lactobacillus*-treated mice, such as tumor necrosis factor-α, IL-6, and IL-1β (59), therefore, the specific role of *Lactobacillus* in DKD needs to be further studied. Taken together, the alterations of gut microbiome in DKD are mainly manifested as the depletion of beneficial bacteria and enrichment of harmful bacteria and potential pathogenic bacteria. Especially, *Hungatella* and *Escherichia* were found predominantly in the comparison between DKD and DM and between DKD and healthy controls. This phenomenon has also been found consistently in the subgroup of clinically diagnosed DKD and biopsy-proven DN, indicating a potential pathogenic mechanism of *Hungatella* and *Escherichia* for DKD.

The genus *R. torques* was demonstrated to be inversely correlated with eGFR of DKD in this review. *R. torques* belongs to mucin-degrading bacteria, which has been suggested to be positively associated with insulin resistance and hyperglycemia (60, 61). The enrichment of *R. torques* was found to have harmful effect on the gut barrier function of elevated lipopolysaccharides translocation, leading to aggravated inflammation in type 2 diabetic rats (62), which might be associated with their renal injury effect.

The advantages of this review is that we systematically searched and screened eligible literature comparing gut microbiome between DKD and non-DKD participants, including diabetes, NDKD, and healthy volunteers. Additionally, all the fecal samples were analyzed using high-throughput sequencing, which may reduce the risk of bias from detection. A recent systematic review also focused on gut bacterial alteration in DKD (15), however, it only compared the differences of gut microbiota between DKD and healthy controls, and studies using bacterial culture and polymerase chain reaction for bacterial analysis were also included in the systematic review. Furthermore, patients with diabetes and other CKD were excluded, which also reduce the bias from participant selection.

Several deficiencies of this review should be considered. First, the definition of DKD was not consistent across the enrolled studies, including biopsy-proven DN and clinically diagnosed DKD, which lead to high heterogeneity of subject selection. Although we have carried out sensitivity analysis and subgroup analysis and obtained relatively stable results, more homogeneous studies are still required to clarify the characteristics of gut microbiota in DKD. Second, some included studies had small sample sizes, while some studies did not match confounding factors between DKD and control group, such as age or sex, which may lead to potential bias. Third, not all of the studies reported the data of all specific outcomes, leading to limited available data, which may result in unstable results that do not fully reflect the underlying differences of gut microbiota. Fourth, most of the studies were conducted in China, and only one cohort was from Europe; therefore, it is still difficult to clarify the differences in intestinal microbiota of DKD patients between different ethnic groups.

## Conclusions

In conclusion, this review indicated alterations of gut microbiota in DKD. Although there were no differences in α-diversity indices between DKD and DM, we found the enrichment of the genera *Hungatella*, *Bilophila*, and *Escherichia* in DKD group. A lower microbial richness and β-diversity were found in DKD compared to healthy controls, more specifically, the phylum *Actinobacteria*, and the genera *Hungatella*, *Butyricicoccus*, *Faecalibacterium*, and *Lachnospira* were proved to be the main differential bacteria. *Faecalibacterium* were significantly depleted in DKD compared to NDKD. Given the potential weakness, substantial heterogeneity, and limited available data, more high-quality evidence is needed to confirm the characteristics of gut microbiota in DKD.

## Data availability statement

The original contributions presented in the study are included in the article/**Supplementary Material**. Further inquiries can be directed to the corresponding authors.

## Author contributions

Conceptualization, YW. Methodology, SH, PC, ZZ, YL, MC, YX, and YW. Software, SH. Funding acquisition, SH and ZZ. All authors contributed to the manuscript writing.

## Funding

This research was funded by National Natural Science Foundation of China, grant number 82274391, 82004263, and the Science and Technology Commission of Shanghai Municipality, China, grant number 20Y21902100, 19401934700.

## Conflict of interest

The authors declare that the research was conducted in the absence of any commercial or financial relationships that could be construed as a potential conflict of interest.

## Publisher’s note

All claims expressed in this article are solely those of the authors and do not necessarily represent those of their affiliated organizations, or those of the publisher, the editors and the reviewers. Any product that may be evaluated in this article, or claim that may be made by its manufacturer, is not guaranteed or endorsed by the publisher.
